# Small Airways Dysfunction and Lung Hyperinflation in Long COVID-19 Patients as Potential Mechanisms of Persistent Dyspnoea

**DOI:** 10.3390/arm92050031

**Published:** 2024-08-23

**Authors:** Angelos Vontetsianos, Nikolaos Chynkiamis, Christina Anagnostopoulou, Christiana Lekka, Stavrina Zaneli, Nektarios Anagnostopoulos, Nikoleta Rovina, Christos F. Kampolis, Andriana I. Papaioannou, Georgios Kaltsakas, Ioannis Vogiatzis, Grigorios Stratakos, Petros Bakakos, Nikolaos Koulouris

**Affiliations:** 1 Rehabilitation Unit, 1st Respiratory Medicine Department, “Sotiria” Hospital, National and Kapodistrian University of Athens, 11527 Athens, Greece; agelvonte@gmail.com (A.V.); christinaanagnosto@gmail.com (C.A.); christiana.lekka@gmail.com (C.L.); stavzaneli@gmail.com (S.Z.); aris.anag@yahoo.gr (N.A.); papaioannouandriana@gmail.com (A.I.P.); georgios.kaltsakas@gstt.nhs.uk (G.K.); ioannis.vogiatzis@northumbria.ac.uk (I.V.); grstrat@hotmail.com (G.S.); petros44@hotmail.com (P.B.); koulnik@med.uoa.gr (N.K.); 2 Thorax Research Foundation, 11521 Athens, Greece; 3 1st Respiratory Medicine Department, “Sotiria” Hospital, National and Kapodistrian University of Athens, 11527 Athens, Greece; nikrovina@med.uoa.gr; 4 Department of Emergency Medicine, “Hippokration” General Hospital of Athens, 11527 Athens, Greece; chkamp77@gmail.com; 5 Lane Fox Respiratory Service, Guy’s and St Thomas’ NHS Foundation Trust, London SE1 7EH, UK; 6 Centre of Human and Applied Physiological Sciences, Faculty of Life Sciences and Medicine, King’s College London, London SE1 1UL, UK; 7 Department of Sport, Exercise and Rehabilitation, Faculty of Health and Life Sciences, Northumbria University Newcastle, Newcastle upon Tyne NE1 8ST, UK

**Keywords:** long COVID-19, small airways disease, lung hyperinflation, dyspnoea

## Abstract

**Highlights:**

**What are the main findings?**

- Long COVID-19 patients may present with small airways dysfunction and lung hyperinflation.
- Lung hyperinflation in patients with long COVID-19 may be associated with persistent dyspnoea.

**What is the implication of the main finding?**

- Small airways disease is prevalent in a group of patients with long COVID-19.
- Early detection of small airways disease and lung hyperinflation in these patients can lead to more targeted treatments and faster recovery rates.

**Abstract:**

Background: Reticulation, ground glass opacities and post-infection bronchiectasis are present three months following hospitalisation in patients recovering from SARS-CoV-2 infection and are associated with the severity of acute infection. However, scarce data exist on small airways impairment and lung hyperinflation in patients with long COVID-19. Aim: To evaluate small airways function and lung hyperinflation in previously hospitalised patients with long COVID-19 and their association with post-COVID-19 breathlessness. Methods: In total, 33 patients (mean ± SD, 53 ± 11 years) with long COVID-19 were recruited 149 ± 90 days following hospital discharge. Pulmonary function tests were performed and lung hyperinflation was defined as RV/TLC ≥ 40%. Small airways function was evaluated by measuring the closing volume (CV) and closing capacity (CC) using the single-breath nitrogen washout technique (SBN_2_W). Results: CC was 115 ± 28% pred. and open capacity (OC) was 90 ± 19. CC was abnormal in 13 patients (39%), CV in 2 patients (6.1%) and OC in 9 patients (27%). Lung hyperinflation was present in 15 patients, whilst the mean mMRC score was 2.2 ± 1.0. Lung hyperinflation was associated with CC (r = 0.772, *p* = 0.001), OC (r = 0.895, *p* = 0.001) and mMRC (r = 0.444, *p* = 0.010). Conclusions: Long COVID-19 patients present with small airways dysfunction and lung hyperinflation, which is associated with persistent dyspnoea, following hospitalisation.

## 1. Introduction

Severe acute respiratory syndrome coronavirus 2 (SARS-CoV-2), responsible for the COVID-19 pandemic, has caused more than 6.5 million deaths worldwide since 2019 [1]. The severity of acute SARS-CoV-2 infection can range from mild disease without evident symptoms to severe respiratory failure requiring intubation associated with increased levels of dyspnoea [2].

According to the NICE definition, long COVID-19 is characterised by symptoms that persist for more than 12 weeks and last for at least 2 months without any other evident causative pathology [3]. The most common symptoms of long COVID-19 include fatigue, dyspnoea, brain fog and post-exertional malaise [3]. Although SARS-CoV-2 can cause thromboembolic episodes during the acute phase of the infection and the development of recurrent venous thromboembolism after hospital discharge has also been described [4,5], pulmonary thromboembolism is not included in the definition of long COVID-19 [3].

While ground-glass opacities and mosaic pattern are the most common abnormalities on thoracic CT scans during the acute phase of the infection [6], reticulation and post-infection bronchiectasis are present three months following recovery [7]. The aforementioned imaging findings may be due to sustained alveolar damage and small airways disease (SAD) [6].

Following SARS-CoV-2 infection, patients present with compromised respiratory function [7,8,9]. A significant number of patients have abnormal values in spirometry for more than 6 weeks after the initial diagnosis [10,11]. Moreover, reduced values of diffusing capacity may be identified for more than 12 months, being one of the most common respiratory function abnormalities following COVID-19 disease [6,7,12]. SARS-CoV-2 viral particles were found by electron microscopy to be deposited in the distal airway mucosal epithelia. This could lead to inflammation of the bronchioles, reduction in their luminal diameter and bronchiolar hyper-responsiveness. The formation of mucosal plugs and bronchial damage in the distal parts of the small airways may result in small airways disease [13]. The evaluation of small airways function is clinically important as it could explain the persistent dyspnoea that is related to decreased exercise capacity in COVID-19 survivors regardless of comorbidities, BMI, smoking status and time since the COVID-19 diagnosis [14].

A few studies assessing small airways function using impulse oscillometry (IOS) exist in this cohort of patients, reporting presence of small airways disease [13,14,15,16]. According to Po-Chun et al., the presence of small airways disease assessed by IOS is associated with static lung hyperinflation, which may be a potential mechanism for post-COVID-19 dyspnoea [17].

However, the IOS technique does not provide measurement for closing volume (CV), which is the lung volume at which small airways start to collapse, and does not assess the uniformity of gas distribution. Compared to other widespread methods to assess small airways function, the single-breath N_2_ washout (SBN_2_W) technique is the only method to directly measure CV and closing capacity (CC) and assess ventilation heterogeneity via the slope of phase III (SIII) [18,19,20]. It is also a feasible and reproducible method for evaluating small airways disease as it is observed in COPD patients [21]. Premature collapse of peripheral airways leads to air trapping and lung hyperinflation resulting in peripheral airway injury, heterogeneity of alveolar ventilation and impaired gas exchange [22]. SBN_2_W has been used to assess small airways function by measuring CV and CC in obstructive airway diseases [22,23]. Nevertheless, the SBN_2_W technique has not been applied in patients with long COVID-19.

The aim of the present study was to assess small airways function and lung hyperinflation in previously hospitalised COVID-19 patients, experiencing symptoms of long COVID-19, and its possible associations with persistent dyspnoea.

## 2. Materials and Methods

### 2.1. Study Design

This retrospective single-centre study assessed small airways function in previously non-ICU hospitalised patients for severe COVID-19, experiencing long COVID-19 symptoms, from January 2022 to December 2022. All participants were adults and met the criteria of long COVID-19 according to NICE 2022 guidelines [3]. Exclusion criteria included the presence of any significant comorbidities including respiratory, cardiac or neuromuscular diseases, active cancer and continued substance abuse. The study was approved by the University Hospital Ethics Committee (Protocol ID-24633).

### 2.2. Respiratory Function Assessment

All participants were evaluated with respiratory function tests at least 3 months following hospital discharge. Spirometry was performed via a metabolic cart (Vmax Encore 22: Sensor Medics, Yorba Linda, CA, USA) using the ‘fast inspiratory manoeuvre technique’ [24]. Static lung volumes were determined using the multiple nitrogen washout technique (Vmax Encore 22 apparatus) and diffusing capacity for carbon monoxide (DLco) was measured via the single-breath technique (Vmax Encore 22 apparatus) [25,26]. Values < 80% predicted for spirometric parameters and diffusion capacity are considered abnormal. In addition, predicted values < 80% and >120% are considered abnormal for static lung volumes. Static lung hyperinflation was defined as RV/TLC ≥ 40% [27].

### 2.3. Single-Breath N_2_ Washout Technique

The single-breath N_2_ washout technique was used to assess small airways dysfunction. After expiration to residual volume, patients performed a slow maximal inspiration followed by a slow (~0.5 L/s) expiratory manoeuvre. Patients continued to expire until the inflection point on the simultaneous record of fractional expired N_2_ concentration (FEN_2_) vs. expired volume was reached. By analysing the inflection point, values for closing volume (CV) and closing capacity (CC) were obtained. Open capacity was defined as the volume between total lung capacity (TLC) and the inflection point [22]. The best-fit line through phase III of the FEN_2_ (%) volume curve (ΔΝ_2_/ΔL) was used to figure the slope of phase III (SIII). The mean of minimum two acceptable tracings was described as the slope of the alveolar gas plateau during phase III. Values > 120% predicted for closing volume and closing capacity were considered abnormal [22,28].

### 2.4. Chronic Dyspnoea Assessment

Chronic dyspnoea was evaluated with the modified Medical Research Council (mMRC) scale [29].

### 2.5. Statistical Analysis

Data are presented as mean ± SD unless otherwise stated. Normal distribution of the data was checked with the Shapiro–Wilk test. Descriptive statistics were used to determine abnormal values in pulmonary function tests (PFTs) and SBN_2_W technique. Patients were categorised in two groups based on the presence or absence of lung hyperinflation (RV/TLC ≥ 40%) [27]. Comparisons between the two groups were performed using two two-tailed independent sample *t*-tests. Correlation analysis was performed using Pearson’s correlation coefficient. Statistical significance was considered when *p* < 0.05. Statistical analysis was performed using the IBM SPSS version 22 statistical software.

## 3. Results

### 3.1. Patient Demographics

Thirty-three patients were included in the analysis, 149 ± 70 days following hospital discharge. Three patients were active smokers (19.3 ± 15.4 pack/years) and eight patients were ex-smokers (32.5 ± 36.9 pack/years). All patients included in the analysis were hospitalised for moderate to severe COVID-19 but without requiring ICU admission. During the acute phase of the disease, all patients received antiviral treatment, and few patients received a low dose of corticosteroids. No cardiac, respiratory or neuromuscular comorbidities, which could affect respiratory function, were reported. Demographic data of the patients are presented in Table 1. Overall, patients presented normal pulmonary function with no obstructive pattern reported via spirometry (Table 1). Chronic dyspnoea assessed via mMRC was 2.2 ± 1.0 (Table 1). Fifteen patients (46%) presented with lung hyperinflation (Table 1).

### 3.2. Pulmonary Function Testing Outcomes

Across all patients, forced mid-expiratory flow (FEF_25–75%_) was 95 ± 33%, CV was 64 ± 28% and CC was 115 ± 28% (Table 2). CC was abnormal in 13 patients (39%), OC in 9 patients (27%), CV in 2 patients (6%) and FEF_25–75%_ in 3 patients (9%). FEF_25–75_ was normal in patients with smoking history, indicating an absence of small airways damage induced by smoking. RV and CC values were greater (both *p* = 0.001), whilst OC was lower (*p* = 0.001) in the lung hyperinflation group (Table 2). In addition, there was a trend for increased volume of phase III in the lung hyperinflation group (*p* = 0.086) (Table 2).

### 3.3. Associations with Chronic Dyspnoea

**Figure 1:**
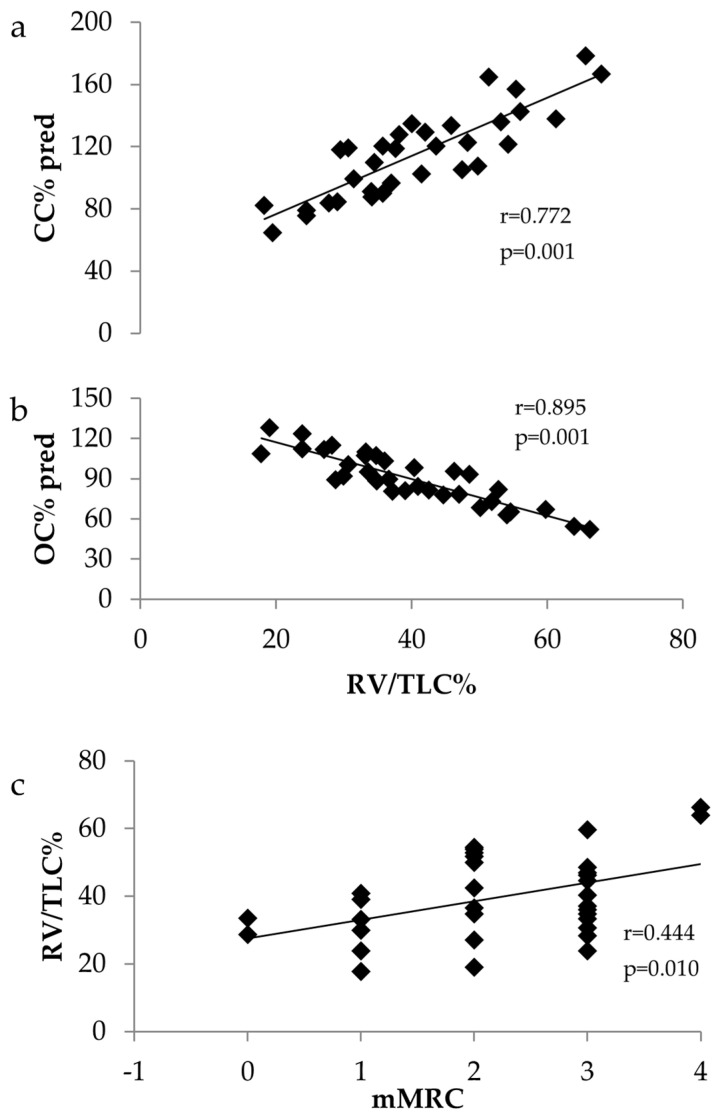
Associations between (**a**) RV/TLC% and CC% predicted, (**b**) RV/TLC% and OC% predicted and (**c**) mMRC and RV/TLC%.

There was a positive correlation between RV/TLC and mMRC (r = 0.444, *p* = 0.010) and CC (r = 0.772, *p* = 0.001) and a negative correlation with OC (r = 0.895, *p* = 0.001) (Figure 1).

## 4. Discussion

To our knowledge, this is the first study investigating small airways function in patients with long COVID-19 using the single-breath N_2_ test. We report that 39% of patients with long COVID-19 presented with small airways dysfunction, as this was defined by increased closing capacity. Premature closure of the small airways was associated with lung hyperinflation, which is a possible pathophysiologic mechanism for post COVID-19 dyspnoea [17].

CT abnormalities were observed in a significant number of patients immediately after recovery and/or hospital discharge [16,30,31] as well as months following infection from SARS-CoV-2 [15,32]. In the chest CT scans of previously hospitalised patients with COVID-19, the mosaic pattern was evident, which was compatible with air trapping and hyperinflation and presented a strong correlation with the RV/TLC ratio [30]. In the same study, it was demonstrated that air trapping persisted for more than 6 months [30]. All the aforementioned studies suggested the presence of SAD in lung imaging. In our study, patients presented with marginally reduced DLco values, which could signify the absence of significant pathologic imaging findings from the lung parenchyma.

Previous studies in patients with long COVID-19 evaluated small airways function via oscillometry [13,14,15,16]. Oscillometry is a technique where a stimulus is applied in the form of random noise in the respiratory system to assess the mechanical properties of the lung passively and measures resistance and reactance of the airways, indirectly indicating small airways dysfunction [33]. In contrast, the SBN_2_W technique that was used in the present study directly measures the lung volume at which small airways start to collapse. CV refers to the amount of remaining gas at the beginning of small airways closure and in healthy subjects, this closure of small airways occurs near the RV with a normal value of 25% of the VC [33].

Indication of small airways impairment was reported at least 3 months following the acute phase of COVID-19 disease via oscillometry [13,14]. Specifically, the increased difference of resistance at 5 and 20 Hz, which is an indicator of small airways disease, was observed in 64% of the long COVID-19 patients 3 months following infection [17]. We reported the presence of SAD measured by increased CC in 39% of patients in our cohort.

According to Bourdin et al., increased values of CV and SIII have been correlated with poor asthma control, a high number of exacerbations, and elevated RV/TLC ratio [34]. In patients with chronic obstructive pulmonary disease, SIII was associated with predicted FEV_1_ and RV/TLC, and it was related to dyspnoea and exercise capacity [21]. Furthermore, the presence of SAD in COPD and asthmatic patients was correlated with more exacerbations and augmented sensation of dyspnoea [35,36]. According to our data, certain SBN_2_W parameters such as CV and SIII presented a weak correlation with the RV/TLC ratio and were not associated with dyspnoea. Nevertheless, lung hyperinflation was associated with CC and it was the main cause for the development of persisting dyspnoea in our cohort.

The causes of dyspnoea following SARS-CoV-2 infection are complex and multifactorial. They can be related to either pulmonary dysfunction or extrapulmonary factors, such as reduced physical activity, hypoxia, malnutrition, systemic inflammation and extrapulmonary immune-mediated damage [37]. Regarding the aetiology of dyspnoea associated with pulmonary dysfunction in long COVID-19, several studies have demonstrated the direct damage to the pneumocytes induced by the virus and the formation of granulation tissue in the small airways [38]. The angiotensin-converting enzyme 2 receptor is utilised by SARS-CoV-2 for viral entry and is mostly located in the smaller airways and the alveoli [39]. This could result in a direct viral effect on the small airways of patients with long COVID-19, which is correlated with lung hyperinflation and persistent dyspnoea [17].

According to Fumagalli et al., forced expiratory volume at the 1st second (FEV_1_) and forced vital capacity (FVC) are reduced 6 weeks following hospitalisation due to SARS-CoV-2 infection [40], whilst they seem to improve approximately 6 months following the acute phase of the disease [10]. Furthermore, Lopes and colleagues reported that FEV1, FVC and FEV1/FVC were abnormal in 34% patients, while FEF_25–75%_ values remained within normal limits, 5 months following hospitalization [13]. In another study, the most common PFTs abnormalities associated with persistent CT abnormalities were DLco, alveolar ventilation (V_A_) and FVC [41].

In line with the aforementioned studies, our results showed normal pulmonary function testing in both groups, except for slightly reduced DLco values, which were not associated with dyspnoea. In our cohort, we demonstrated that SBN_2_W is more sensitive in detecting SAD compared to using FEF_25–75%_ in long COVID-19 patients with preserved lung function. Lung hyperinflation was correlated with small airways dysfunction in patients with long COVID-19.

### 4.1. Study Limitations

Our study has several limitations. Initially, the subjects in our cohort were infected with the alpha and delta variants of SARS-CoV-2 and were prescribed antiviral and anti-inflammatory treatment during their hospitalisation. Due to the small sample size, our study has limited generalizability and subgroup analyses were not possible. Furthermore, the effects of different treatments for COVID-19 on small airways were not evaluated in the present study, and thus still remain unknown. Another limitation of the present study is that no follow-up visits were performed, and thus detecting any potential changes in small airways dysfunction, lung hyperinflation and post COVID-19 breathlessness over time was not possible. Data from chest CT scans were not available and no certain imaging patterns could be identified. Additionally, we are lacking data on airways function and lung hyperinflation prior to COVID-19 in our cohort. Randomised controlled trials should be performed with long follow-up periods to examine the duration of post COVID-19 dyspnoea and whether management of small airways disease could result in symptom amelioration.

### 4.2. Clinical Implications

In line with the current literature, our results demonstrate the effects of small airways dysfunction and lung hyperinflation on dyspnoea and provide new insights on the importance of the small airways disease in the pathophysiology of long COVID-19. The SBN_2_W technique is a feasible and effective test in detecting small airways dysfunction in patients with long COVID-19. Although long COVID-19 patients often present with small airways disease and lung hyperinflation, which are associated with persistent dyspnoea and decreased exercise capacity, little is known about the management of these patients. It is well documented that small airways disease is associated with worse spirometry results, poorer health status and quality of life in patients with COPD, making the small airways an important treatment target [42]. The use of inhaled long-acting bronchodilators or oral and/or inhaled corticosteroids three months after COVID-19 improves quality of life 15 months following the acute phase of the disease [43]. Thus, it is possible that administering bronchodilation treatment for the management of patients with long COVID-19 and small airways dysfunction might be beneficial. Nevertheless, more studies are required to establish the beneficial effect of bronchodilation administration in these patients.

## 5. Conclusions

Small airways dysfunction assessed via the single-breath nitrogen washout technique was associated with lung hyperinflation in previously hospitalised long COVID-19 patients. Moreover, lung hyperinflation was present at least three months after the acute infection, which is in line with other studies, and it was associated with post-COVID-19 dyspnoea. Hence, future studies should confirm the presence of small airways disease in larger groups of patients and focus on interventions to manage lung hyperinflation, relieving their symptomatology and improving their quality of life.

## Figures and Tables

**Table 1:** Demographic characteristics in all patients and comparison between patients with and without lung hyperinflation.

	All	RV/TLC < 40%	RV/TLC ≥ 40%	*p* Value
Gender (m/f)	14/19	10/8	4/11	0.223
Age (years)	53.4 ± 11.4	51.1 ± 12.2	56.1 ± 10.1	0.219
Height (cm)	166 ± 12	166 ± 12	165 ± 13	0.755
Weight (kg)	78 ± 18	81.2 ± 20.0	73.8 ± 15.6	0.247
BMI (kg·m^2^)	28.1 ± 5.4	29.0 ± 5.0	27.0 ± 6.0	0.292
Time from discharge (days)	149 ± 70	148 ± 93	149 ± 92	0.962
mMRC score	2.2 ± 1.0	1.9 ± 1.1	2.6 ± 0.8	0.045
FEV_1_ (%pred)	100 ± 19	100 ± 19	100 ± 20	0.914
FVC (%pred)	99 ± 20	97 ± 18	101 ± 22	0.604
FEV1/FVC (%)	84 ± 6	85 ± 5	83 ± 7	0.508
TLC (%pred)	94 ± 27	83 ± 14	108 ± 31	0.004
RV/TLC (%)	40 ± 12	31 ± 6	51 ± 8	0.001
FRC (%pred)	98 ± 44	77 ± 23	123 ± 49	0.001
DLco (%pred)	78 ± 23	78 ± 19	77 ± 27	0.904

mMRC: modified Medical Research Council Dyspnoea Scale, FEV_1_: forced expiratory volume at the 1st second, FVC: forced vital capacity, RV: residual volume, TLC: total lung capacity, FRC: functional residual capacity, DLco: diffusion capacity for carbon monoxide.

**Table 2:** Small airways function in patients with and without lung hyperinflation.

	All	RV/TLC < 40%	RV/TLC ≥ 40%	*p* Value
FEF_25–75_ (%pred)	95 ± 33	93 ± 27	96 ± 39	0.823
RV (%pred)	113 ± 66	76 ± 25	158 ± 72	0.001
CV (%pred)	64 ± 28	68 ± 27	58 ± 30	0.314
CC (%pred)	115 ± 28	99 ± 20	135 ± 23	0.001
V_III volume (%pred)	95 ± 17	91 ± 10	101 ± 23	0.086
N_2_-slope (%N_2_/L)	1.00 ± 0.68	1.06 ± 0.60	0.93 ± 0.78	0.580
N_2_-slope (%pred)	71 ± 52	76 ± 43	65 ± 63	0.567
OC (%pred)	90 ± 19	102 ± 14	76 ± 14	0.001

FEF_25–75_: forced mid-expiratory flow, RV: residual volume, TLC: total lung capacity, CV: closing volume, CC: closing capacity, V_III: slope of phase III, N_2_: nitrogen, OC: open capacity.

## Data Availability

The data presented in this study are available upon request from the corresponding author.
